# The SAPS 3 score as a predictor of hospital mortality in a South African tertiary intensive care unit: A prospective cohort study.

**DOI:** 10.1371/journal.pone.0233317

**Published:** 2020-05-21

**Authors:** Elizabeth van der Merwe, Jacinto Kapp, Sisa Pazi, Ryan Aylward, Minette Van Niekerk, Busisiwe Mrara, Robert Freercks

**Affiliations:** 1 Adult Critical Care Unit, Livingstone Tertiary Hospital, Port Elizabeth, South Africa; 2 Walter Sisulu University, Mthatha, South Africa; 3 Department of Statistics, Nelson Mandela University, Port Elizabeth, South Africa; 4 Division of Nephrology and Hypertension, Livingstone Hospital, Port Elizabeth, South Africa; 5 Division Nephrology and Hypertension, Department of Medicine, University of Cape Town, Cape Town, South Africa; Azienda Ospedaliero Universitaria Careggi, ITALY

## Abstract

**Background:**

No African countries were included in the development of the Simplified Acute Physiology Score 3 (SAPS 3). This study aimed to assess the performance of the SAPS 3 as a predictor of hospital mortality in patients admitted to a multi-disciplinary tertiary intensive care unit (ICU) in South Africa.

**Methods:**

A prospective cohort study was undertaken in a tertiary single-centre closed multidisciplinary ICU with 16 beds over 12 months in 2017. First time admissions 12 years and over were included. Exclusions were patients who died within six hours of admission, incomplete data sets and unknown outcome after ICU discharge. Demographic data, clinical admission data and co-morbidities were recorded. The SAPS 3 score was calculated within the first hour of ICU admission. The highest Sequential Organ Failure Assessment score, vasopressor use, mechanical ventilation requirements and details of acute kidney injury, if present, were recorded. Discrimination of the model was evaluated using an area under the receiver operating characteristic curve (AUROC) and calibration by the Hosmer-Lemeshow (HL) Goodness of Fit Test (Ĉ and Ĥ statistic). The observed versus the SAPS 3 model predicted mortality ratios were compared and the standardized mortality ratio (SMR) was calculated.

**Results:**

A total of 829 admissions with a mean SAPS 3 (SD) of 48.1 (16) were included. Of patients with a known human immunodeficiency virus (HIV) status, 32,4% were positive. The ICU and hospital mortality rates were 13.3% and 21.4% respectively. The SAPS 3 model had a AUROC of 0.796 and HL Ĉ and Ĥ statistics were 12.1 and 11.8 (p-values 0.15 and 0.16). The SMR for the model was 1.002 (95%CI: 0.91–1.10). The mortality of 41% for the subgroup with sepsis/septic shock was higher than predicted with a SMR of 1.24 (95% CI 1.11–1.37).

**Conclusions:**

The SAPS 3 model showed good calibration and fair discrimination when applied to the cohort. The SAPS 3 model can be used to describe the case mix in this African ICU with a high incidence of HIV. Ongoing efforts should be made to improve outcomes of septic patients.

## Introduction

Several prognostic scoring systems have been developed to predict the hospital mortality of intensive care unit (ICU) patients [1]. Commonly used models like the Acute Physiology and Chronic Health Evaluation II and the Simplified Acute Physiology Score II [2] are used to describe the case mix for research and clinical auditing purposes [1]. Since the development of these models, there have been significant changes in the prevalence of diseases and in diagnostic and therapeutic methods. The Simplified Acute Physiology Score 3 (SAPS 3) is a more modern mortality prediction model which has been developed using a larger cohort, including for the first time, cohorts from outside North America and Europe and taking advantage of newer computer-intensive methods of analysis [3]. However, no African ICU was part of the development cohort and although data on human immunodeficiency virus (HIV) status was not recorded, less than 0.5% of participants had the acquired immunodeficiency syndrome (AIDS) [4]. Also, the incidence and prevalence of HIV in the participating countries is known to be considerably lower than in Africa [5].

The South African public health care system services 84% of the population but accounts for less than 55% of the total health care expenditure [6, 7]. The ICU bed to total hospital bed ratio in South Africa is 1.7% in the public sector compared with 8.9% in the private sector [8]. Depending on the level of care, the ratio in high income countries tends to be between 2.5% and 10% [9, 10]. This shortage of ICU beds has therefore resulted in the need for triage in South African public health care sector critical care units [11, 12]. Objective data regarding ICU outcomes, which would include accurate case mix description with a modern severity score, is therefore essential for resource allocation and to inform local patient triage guidelines [8, 13].

The performance of a prediction model is evaluated by the calibration and discrimination of the model and by calculating the standardised mortality ratio (SMR). The SMR is amongst the safety and quality indicators that an ICU should apply to evaluate quality of care [14].

This study aimed to assess the performance of the SAPS 3 in its ability to predict hospital mortality amongst critically ill patients of different case mixes admitted to a tertiary ICU in South Africa.

## Methods

### Study methods

The study design was a prospective observational study conducted in a closed multi-disciplinary closed, 16 bed tertiary ICU with full time onsite resident cover, full time specialist cover and a registered nurse to patient ratio of 3:4. Livingstone Tertiary Hospital serves approximately 1.6 million people from an area of 60 000 km2. The data was extracted from the database of an observational ICU study: Acute kidney injury in critically ill patients in a tertiary intensive care unit in the Eastern Cape, South Africa (the LivAKI study) which included 875 ICU admissions to Livingstone Tertiary Hospital (LTH) Adult ICU [15]. All first time ICU admissions 12 years and older, admitted from the 3rd of January 2017 to the 2nd January 2018 were included. Exclusion criteria were patients who arrived moribund and died within six hours of admission (considered inappropriate ICU referrals), patients admitted to step down beds for drug infusions, patients with incomplete data sets, e.g. unknown hospital outcome, patients who were readmitted to ICU, brain dead patients admitted for organ donation and patients with end stage kidney disease who were found ineligible for long term renal replacement therapy due to resource limitations.

The SAPS 3 score has 20 variables including physiological parameters, patient characteristics before ICU admission and circumstances surrounding ICU admission. Values from the first hour after admission are used. Data was collected and processed to formulate the SAPS 3 predicted mortality rates, using the formula recommended by Moreno et al [4]. Demographic data, details related to admission and co-morbidities were recorded. For patients known with HIV, treatment status with highly active antiretroviral therapy (HAART), a premorbid CD4 count and viral load were recorded where available. The Sequential Organ Failure Assessment (SOFA) score was calculated 24 hours after admission and every third day thereafter, or sooner if the patient’s condition deteriorated [16]. The highest score during the patient’s admission was recorded. Vasopressor use, mechanical ventilation requirements, the presence of acute respiratory distress syndrome (ARDS) and acute kidney injury (AKI), were also recorded. AKI was diagnosed and staged according to the Kidney Diseases Improving Global Outcomes (KDIGO) definition [17] and ARDS according to the Berlin definition [18]. Sepsis and septic shock was defined using the Sepsis-3 criteria [19]. All score sheets were reviewed independently by the researchers of the LivAKI study to check for any data errors. ICU and hospital mortality were recorded.

Data was exported from the Research Electronic Data Capture (REDCap) hosted at the University of Cape Town [20] and analyzed with RStudio, 2019 (Version 3.6.1; http://www.r-project.org). Continuous data were tested for normality using the Kolmogorov-Smirnov, Shapiro-Wilk, Anderson-Darling and Pearson's chi-squared tests. Normally distributed data were reported as means (standard deviation) and skewed data as medians (interquartile range). Discrete data were presented as numbers (percentages). The student’s t-test and the Mann-Whitney U test were used to compare continuous data and the chi-square and Fischer’s exact tests were used for discrete data, as appropriate. Calibration of the model was assessed by the Hosmer-Lemeshow (HL) ‘goodness of fit’ test and visually represented as a calibration curve [21] with a p-value greater than 0.05 implying that the prediction model has non-significant departure from perfect calibration. HL Ĉ and Ĥ statistics were calculated. If fixed deciles are used it is called the HL Ĥ statistic and if naturally occurring deciles (equal number of patients per group) are used, it is called the HL Ĉ statistic. Discrimination was assessed by the AUROC. The discrimination of the prognostic model was considered good if the AUROC was > 0.8, moderate if between 0.6 and 0.8, and no better than chance when < 0.6 [22, 23]. The SMR was computed by dividing the observed number of hospital deaths by the SAPS 3 predicted number of deaths. In all cases, the level of significance was 5%.

#### Ethical standards

The study was approved by the Walter Sisulu University Human Research Ethics Committee (REF: 120/2018). Datasets are accessible from the Mendeley Data public repository available at http://dx.doi.org/10.17632/7f6yxz2d4c.1.

## Results

During the study period, there were 875 admissions. Forty-six were excluded (Table 1), leaving 829 admissions for inclusion in the analysis.

**Table 1:** Number of participants excluded from the final sample due to exclusion criteria.

**Total cohort**	875
Readmission	8
Incomplete data sets	4
Deceased within 6 hours of admission	15
Certified brain dead (admission for organ donation)	3
Admission for chemotherapy	3
End stage kidney failure, not for dialysis	1
Hospital outcome unknown	12
**Total exclusions**	**46**
**Final number of participants analysed**	**829**

Baseline characteristics of the cohort are displayed in Table 2. The ICU and hospital mortality was 13.3% and 21.4% respectively. The median ICU length of stay was 3 days (IQR 1–6). 24,6% of patients stayed 7 days or more and this subgroup had a hospital mortality of 31%. Of all admissions, 30.8% were medical, 66.4% surgical and 2.8% obstetrics-related. The surgical category comprised of general surgical, trauma, orthopaedic, neurosurgical and vascular cases which were sub-divided into elective surgical admissions (19.8%) and emergency surgical admissions (80.2%). The most common diagnoses admitted were assault (14%), motor and pedestrian vehicle accidents (12%), acute abdomen (10%), pneumonia (6%; including cases later identified as pulmonary tuberculosis) and self-inflicted drug/toxin overdose (3.5%).

**Table 2:** Baseline characteristics of participants.

Characteristic		All patients N = 829	Survivors 652 (78.6%)	Non-survivors 177 (21.4%)	p-value
Mean age years (SD)		42.6 (16.7)	41.5 (16.6)	46.6 (16.7)	< 0.001
Male n (%)		490 (59.0)	386 (59.2)	104 (58.8)	0.915
Type of admission n (%)	Medical	255 (30.8)	200 (30.7)	55 (31.6)	0.915
	Surgical–elective	109 (13.2)	99 (15.2)	10 (5.6)	< 0.001
	Surgical–emergency	442 (53.3)	336 (51.5)	106 (59.9)	0.048
	Obstetrics and gynaecology	23 (2.8)	17 (2.6)	6 (3.4)	0.570
Race	Black	481 (58.0)	378 (58.0)	103 (58.2)	0.959
	Mixed	227 (27.4)	177 (27.2)	50 (28.3)	0.771
	Caucasian	114 (13.8)	91 (14)	23 (13)	0.742
	Indian	5 (0.6)	4 (0.6)	1 (0.6)	*
	Other	2 (0.2)	2(0.3)	0	*
Admission SOFA score (IQR)		4 (1–6)	3 (1–5)	7 (4–10)	<0.001
Highest SOFA score (IQR)		4 (2–7)	3 (1–5)	9 (6–12)	<0.001

*Sample size too small for comparison. SD: Standard Deviation, IQR: Interquartile Range, SOFA: Sequential Organ Failure Assessment.

The hospital mortality for surgical and medical emergencies was 24.0% and 21.6% respectively. Obstetric and gynaecological (O&G) emergencies had a mortality of 26.0%. For the surgical subgroup admitted with trauma (trauma cases overlap with orthopaedic, surgical and neurosurgical emergencies) the mortality was 23.7%. Ten patients died following elective surgery: 4 neurosurgical cases, 4 vascular surgical patients and 2 general surgical cases.

The mortality rate for subgroups known to be at high risk for non-survival, are summarized in Table 3.

**Table 3:** Subgroups at high risk for non-survival.

Diagnostic groups	Number of patients	Survivors with condition	Non-survivors with condition	p-value	SMR (95%CI)	Mortality (%)
Sepsis or septic shock n (%)	251 (30.3)	148 (22.7)	103 (58.2)	<0.001	1.24 (1.11–1.37)	41.0%*
ARDS n (%)	35 (4.2)	22 (3.4)	13 (7.3)	0.020	0.91 (0.60–1.21)	37.1%
AKI n (%)	485 (59.0)	332 (51)	153 (86)	<0.001	1.10 (1.00–1.20)	31.6%
Mechanical ventilation n(%)	444 (53.6)	293 (45)	151 (85.3)	<0.001	1.12 (1.02–1.23)	34.0%
Requiring vasopressor n (%)	209 (25.2)	97 (14.9)	112 (63.0)	<0.001	1.31 (1.19–1.43)	53.6%
ICU days >7 days n (%)	175 (21.1)	119 (18.3)	56 (31.6)	0.001	1.16 (0.97–1.30)	32.0%

*Mortality for septic shock was 54%. SMR: Standardised mortality ratio, ARDS: Acute Respiratory Distress Syndrome, AKI: Acute Kidney Injury, ICU: Intensive care unit.

The HIV status was known in 56.1% of admissions, of which 32.4% were positive (Table 4). A significantly higher mortality was observed in patients who tested positive for HIV (27.8%) compared to known HIV negative patients (18.4%), p = 0.03. However, HIV positive patients also had both a significantly higher mean SAPS 3 score (58.15 vs 53.31, p = 0.007) and admission SOFA score (8 vs 6, p = 0.01). On multivariate analysis of the patients with a known HIV status, HIV positive patients were more likely to have higher SAPS scores (p = 0.012) and a diagnosis of sepsis any time during their stay (p = 0.030). The specific SAPS variables where HIV patients scored higher than HIV negative patients, were reason for admission (unplanned ICU admission), trauma related surgery and nosocomial acute infection at ICU admission (p< 0.05 for all). Additionally, HIV positive patients had a higher serum creatinine, lower white cell count, lower pH and lower platelet counts compared to HIV negative patients (p< 0.05 for all). Patients with unknown HIV status had a mortality percentage comparable to the overall cohort (21.2%). Neither HAART, premorbid CD4 count nor viral load log were associated with mortality (Table 4).

**Table 4:** HIV related data.*

Patient characteristic	HIV status known (n = 465)	Survivors 365 (78.5%)	Non-survivors 100 (22.5%)	p-value
HIV positive n (%)	151 (32.5)	109 (30)	42 (42)	0.020
Median premorbid CD4 (IQR) n = 51	309 (141–483)	331 (186–503)	167 (118–337)	0.170
Median viral load log (IQR) n = 16	2 (1.92–3.42)	2 (1.92–3.42)	2 (1.95–3.31)	0.951
Receiving HAART n (%)	97 (64.2)	73 (67)	24 (57.1)	0.259

* Analysis restricted to those with known HIV serostatus. IQR: Interquartile Range, HAART: Highly active antiretroviral therapy

**Fig 1:**
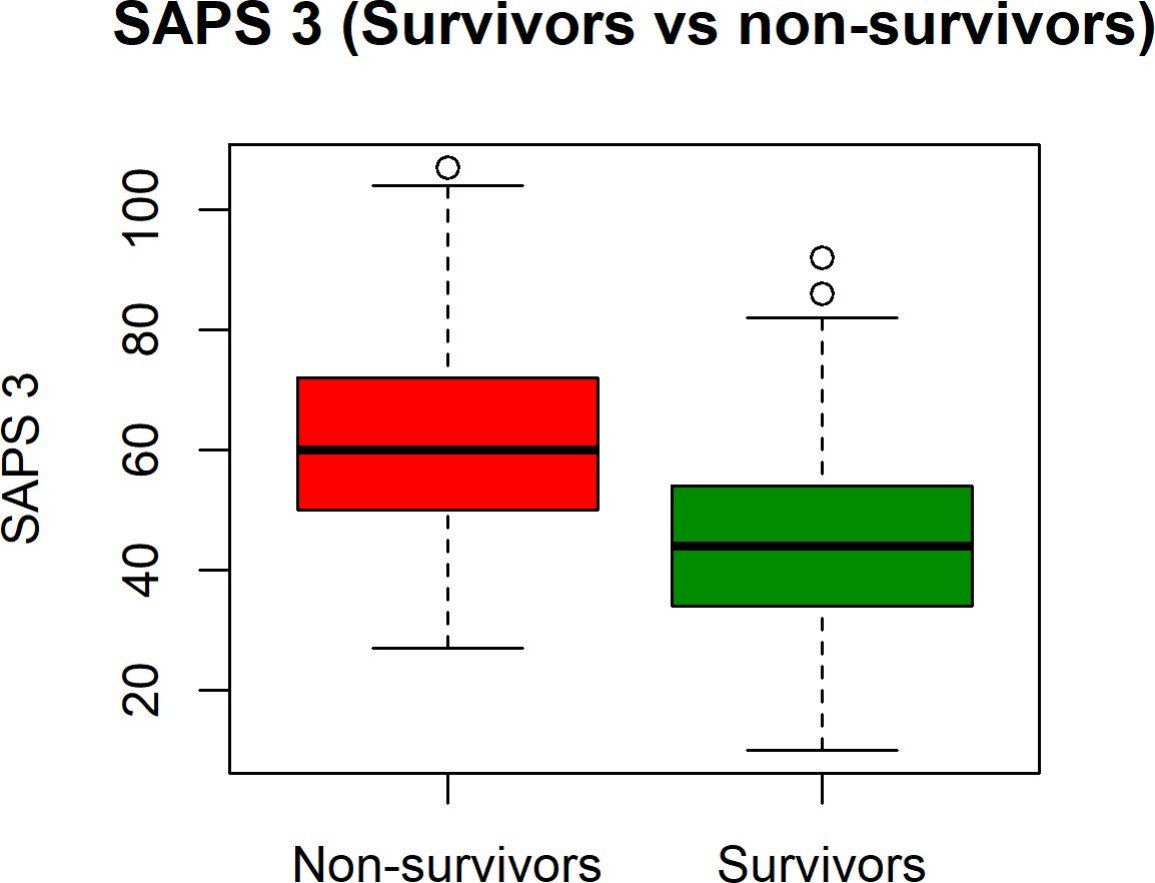
Box and whisker plot summarizing the mean SAPS 3 scores of hospital survivors and non-survivors.

The mean SAPS 3 score was 48.1 and was significantly higher for non-survivors (61.58, SD 15) than for survivors (44.4, SD 14.2), p < 0.001 (Fig 1). There were no survivors with a SAPS 3 score over 92.

**Fig 2:**
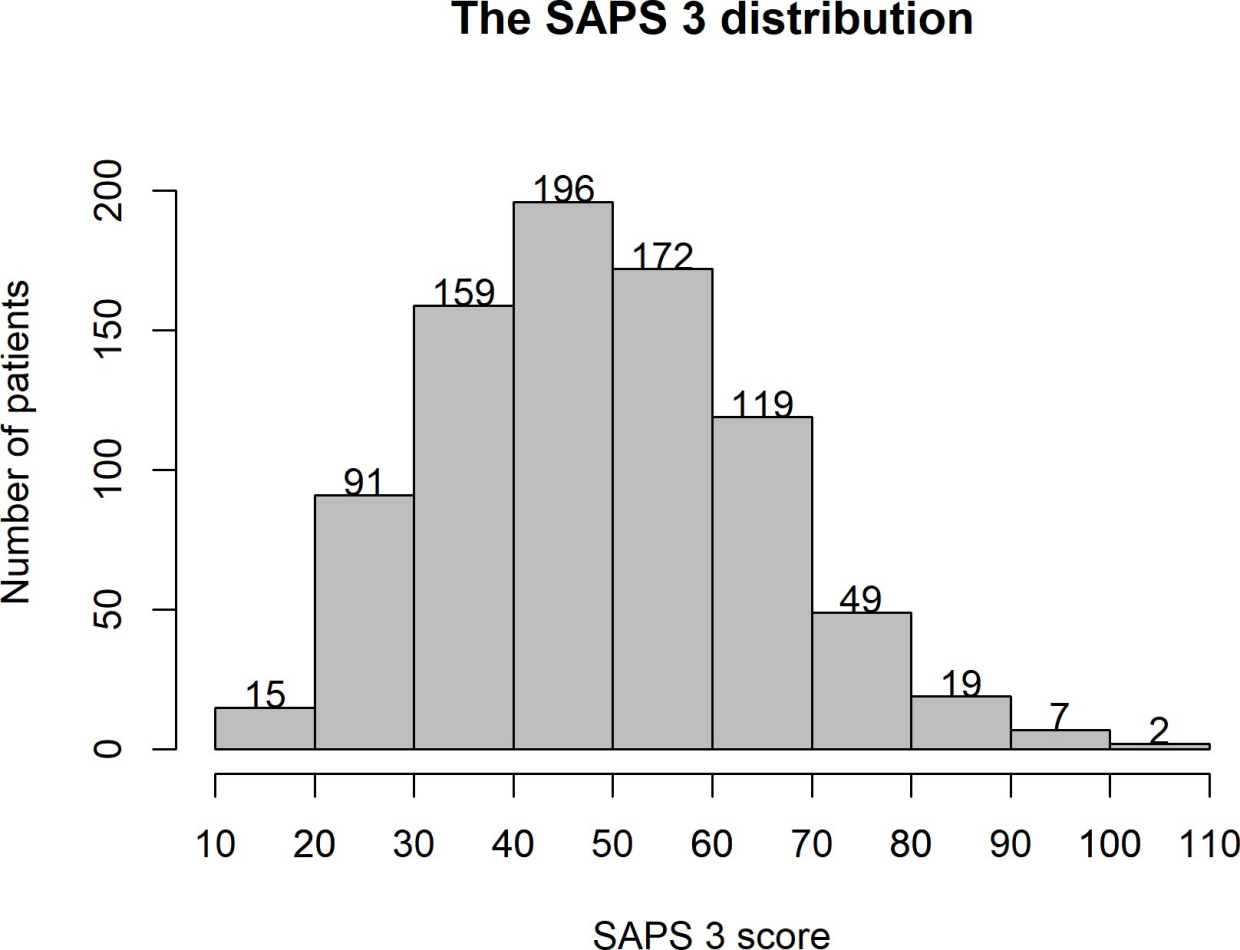
The SAPS 3 distribution.

The distribution of SAPS 3 scores is shown in Fig 2. The highest number of admissions (23.6%) had a SAPS 3 score between 40 and 50 (predicted hospital mortality 6–17%).

**Fig 3:**
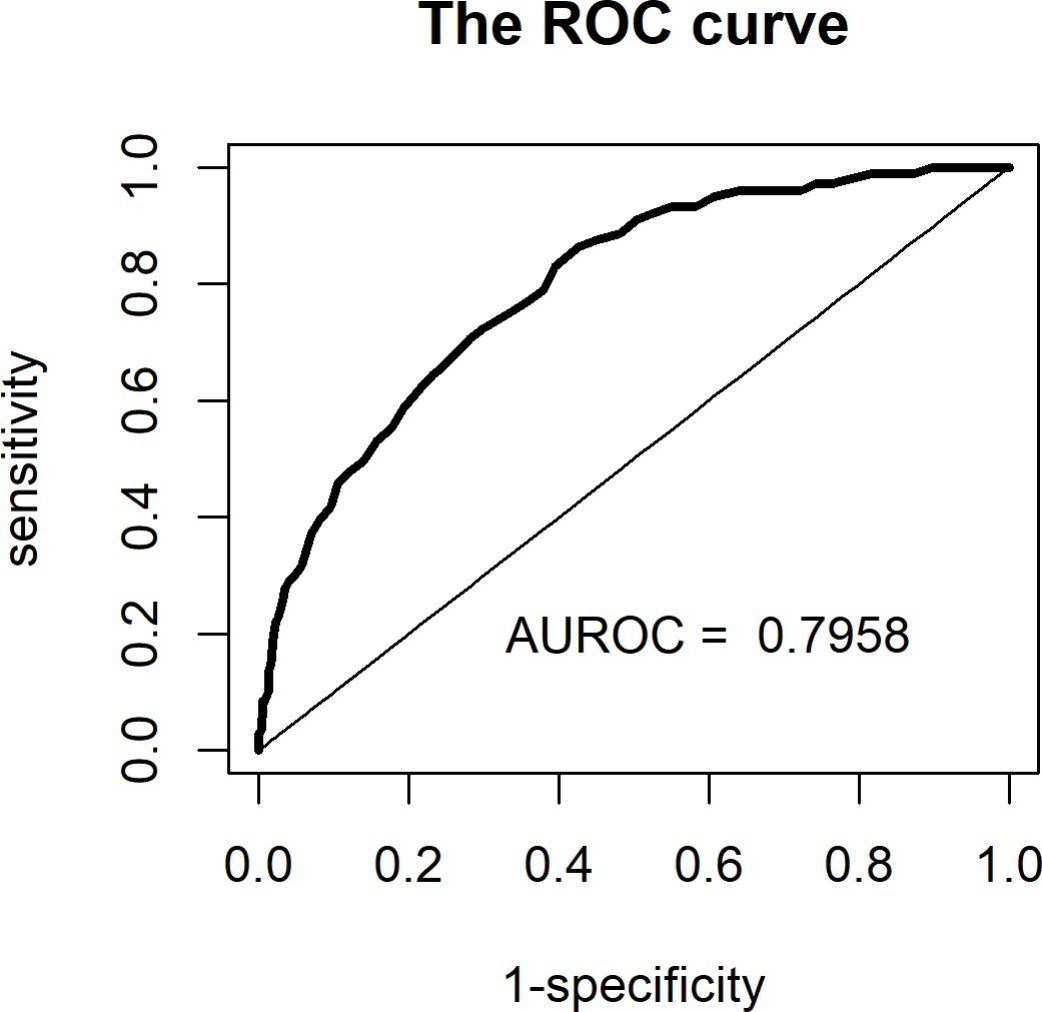
Receiver operator characteristic curve for the SAPS 3 model.

The AUROC curve was 0.796 (Fig 3) and indicates moderate discrimination of the model. A SAPS 3 score of more than 53 predicted non-survival with a sensitivity of 75% and specificity of 83%.

**Fig 4:**
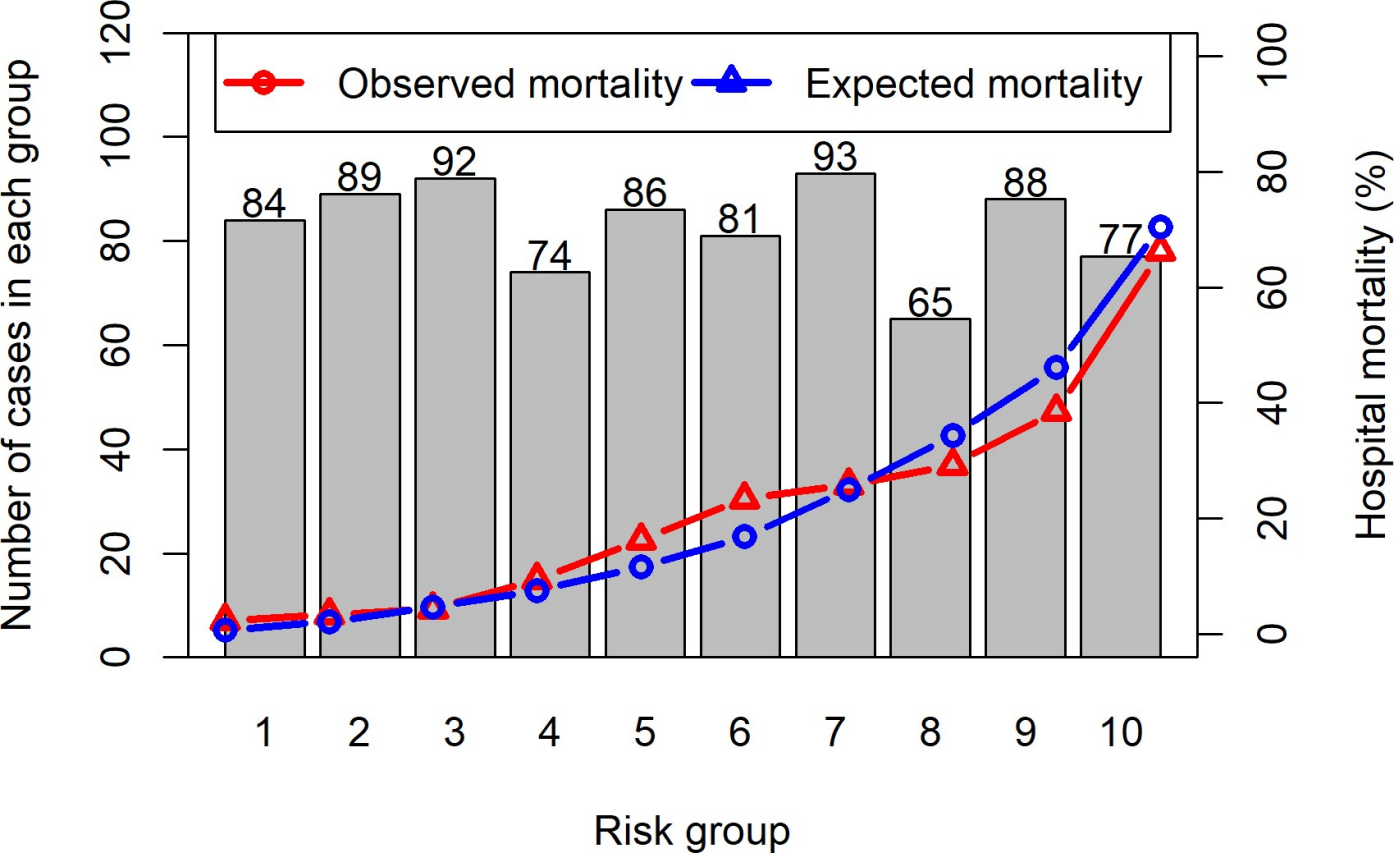
Calibration curve (Hosmer-Lemeshow Ĉ statistic) for the overall sample.

The HL Ĉ and Ĥ statistics were 12, 1 and 11,8 and both showed non-significant departure from the predicted outcomes of the model (p-values 0.15 and 0.16 respectively). The SAPS 3 calibration curve (HL Ĉ statistic) is shown in Fig 4.

The SMR was 1.002 (95%CI: 0.91–1.1). The SMRs for certain subgroups at high risk are shown in Table 3.

## Discussion

To our knowledge this is the first prospective study to test the performance of the SAPS 3 score in an African ICU. The SAPS 3 model showed good calibration and fair discrimination in predicting hospital mortality in this tertiary adult ICU. Calibration measures how well the estimated mortality of the prediction model correlates with observed mortality in a cohort over a wide range of probabilities [24] while discrimination reflects the ability to distinguish between survival and non-survival in an individual [21]. Analysis of discrimination is only valid if the model has first been shown to calibrate well [25].

The SMR is also a measure of calibration and allows for comparison between the observed and predicted number of deaths. The SMR in this cohort of 1.002 (95% CI: 0.91–1.10) indicates that overall, the hospital mortality is comparable to the original cohort where the SAPS 3 score was developed and validated. In a 2014 systematic review of SAPS 3 validation studies, most studies from higher income countries now report SMRs lower than 1.0, while studies from South and Latin America mostly had SMRs of 1.0 [26]. The SAPS 3 model did not perform as well in all subgroups in our cohort (Table 3). Those with sepsis/septic shock had a mortality of 41% which was 24% higher than expected (SMR of 1.24; (95% CI 1.11–1.37)). While the mortality rate of 54% for septic shock was higher than the 42.3% reported by the Surviving Sepsis Campaign database [19] and 38% reported by a recent systematic review from North America and Europe [27], it is in keeping with, or lower than, the mortality seen in cohorts from other upper middle income countries e.g. China (53%) [28], Brazil (60%) [29] and Turkey (70%) [30]. It has been recognised that early appropriate antibiotics and early appropriate resuscitation are likely to improve outcomes in sepsis and septic shock [31]. Geographical, socio economic, educational and health system factors may contribute to patients with sepsis not accessing these interventions timeously in lower and middle income countries as compared to high income countries. This needs further investigation.

The high percentage of surgical compared to medical patients (69.2% versus 30.7%), reflects the well-described health care burden of motor vehicle accidents and interpersonal violence in South Africa [32–34]. The low number of O&G admissions (2.8%) is explained by the presence of a separate O&G high care; only cases requiring ventilation or dialysis are transferred to ICU. The relatively low percentage of elective admissions is a reflection of the fact that emergency admissions are often triaged over planned surgery in South Africa when there are not enough ICU beds available [11]. The elective cases who did not survive were largely complicated vascular and neurosurgical cases.

The HIV seropositive rate in our ICU cohort is consistent with the known background HIV prevalence of 25.2% in adults aged 15–49 in this province [35]. The LTH ICU admission policy requires triaging of all patients, taking perceived benefit of ICU admission, frailty and performance status into consideration. While a higher mortality was observed in HIV positive as compared to HIV negative patients, this could be explained by their significantly higher severity of illness, as indicated by higher SAPS 3 scores and a more frequent diagnosis of sepsis/septic shock on multivariate analysis. There was a trend for HIV positive non-survivors to have lower premorbid CD4 count. Although 64% of patients were on ARV’s, only 34% of (51/151) HIV positive patients had a recent pre-morbid CD4 count available. Reasons for this may include poor access to care and/or poor patient adherence to follow-up. Similarly, only 11% of patient had a HIV viral load result available. While the policy is to offer HIV testing to all awake patients, implementation of this needs to be improved in view of the 56% test rate in the cohort.

The marked difference between the hospital and ICU mortality could be due a variety of factors including end-of-life care. When treatment escalation is considered non-beneficial, palliation occurs in the LTH ICU, with the exception of patients who do not recover from neurological injury and are breathing on their own. Indeed, on scrutiny, 30 (44.7%) of the 67 patients who died after ICU discharge had a diagnosis of traumatic brain injury, stroke or hypoxic brain injury and were discharged with an unfavourable Glasgow outcome score for palliation. A further 11 (16.4%) who died in the ward after discharge had uncontrolled HIV disease (high viral loads, not on ARV’s, failed ARV regimens and/or low premorbid CD4 counts). Other reasons for demise in hospital after ICU discharge may be due to premature discharge from ICU, medical care in wards and resource limitations. There is considerable pressure on ICU beds in SA [11] and this may prompt early discharge from ICU to accommodate patients who requires organ support.

The study has some limitations. The original SAPS 3 score was validated in patients 16-years and older. In South Africa, due to the population’s age distribution, admission criteria for adult ICU’s mostly include patients from 12 and older. Twenty patients aged 12 to 15 years old, were included in our analysis and this reflects the reality of local admission criteria. Re-analysis of the AUROC, HL Ĉ and Ĥ statistics and SMR, excluding the patients younger than 16, did not change the findings of this study. Although this is the first study to validate SAPS 3 in an African country, the study was conducted in a single-centre ICU and the results may not be applicable to other ICU’s in South Africa of Africa. No statistical test was used to analyse variance between capturers, however potential bias related to data gathering was limited through researchers checking all captured data sheets independently. The vital status of twelve patients who were down-referred to secondary hospitals could not be confirmed and they had to be excluded from the final cohort. Strengths include the large number of patients included, the prospective nature of the study as in the original SAPS 3 validation studies, the use of hospital mortality rather than ICU mortality as outcome measure and the inclusion of a large number of HIV positive individuals.

The discrimination of the SAPS 3 model for local use can be improved through regional customization [14]. The SAPS 3 provides customized equations for seven different regions of the world, which theoretically improves its accuracy but there is no customised version for Africa. The calibration of prediction models is very sensitive to cohort size and few institutions have enough data to develop robust local models [26]. A multicentre South African or African study would address bias with regards to case mix and a large multicentre trial would be required to validate the SAPS 3 score for general use in the region.

## Conclusions

The SAPS 3 model showed good calibration and fair discrimination and can be used to describe the case mix in this African ICU with a high incidence of HIV. Ongoing efforts should be made to improve outcomes of septic patients. Further multicentre studies would be required to validate this model for regional use and customization.
